# Green Synthesis of Bioinspired Nanoparticles Mediated from Plant Extracts of *Asteraceae* Family for Potential Biological Applications

**DOI:** 10.3390/antibiotics12030543

**Published:** 2023-03-08

**Authors:** Juhi Puthukulangara Jaison, Balamuralikrishnan Balasubramanian, Jaya Gangwar, Nilina James, Manikantan Pappuswamy, Arumugam Vijaya Anand, Naif Abdullah Al-Dhabi, Mariadhas Valan Arasu, Wen-Chao Liu, Joseph Kadanthottu Sebastian

**Affiliations:** 1 Department of Life Sciences, School of Sciences, Christ University, Bangalore 560029, India; 2 Department of Food Science and Biotechnology, College of Life Science, Sejong University, Seoul 05006, Republic of Korea; 3 Department of Human Genetics and Molecular Biology, Bharathiar University, Coimbatore 641046, India; 4 Department of Botany and Microbiology, College of Science, King Saud University, P.O. Box 2455, Riyadh 11451, Saudi Arabia; 5 Department of Animal Science, College of Coastal Agricultural Sciences, Guangdong Ocean University, Zhanjiang 524088, China

**Keywords:** *Asteraceae* family, green synthesis, nanoparticle, phytochemicals, characterization, biological activity, toxicity

## Abstract

The *Asteraceae* family is one of the largest families in the plant kingdom with many of them extensively used for significant traditional and medicinal values. Being a rich source of various phytochemicals, they have found numerous applications in various biological fields and have been extensively used for therapeutic purposes. Owing to its potential phytochemicals present and biological activity, these plants have found their way into pharmaceutical industry as well as in various aspects of nanotechnology such as green synthesis of metal oxide nanoparticles. The nanoparticles developed from the plants of *Asteraceae* family are highly stable, less expensive, non-toxic, and eco-friendly. Synthesized *Asteraceae*-mediated nanoparticles have extensive applications in antibacterial, antifungal, antioxidant, anticancer, antidiabetic, and photocatalytic degradation activities. This current review provides an opportunity to understand the recent trend to design and develop strategies for advanced nanoparticles through green synthesis. Here, the review discussed about the plant parts, extraction methods, synthesis, solvents utilized, phytochemicals involved optimization conditions, characterization techniques, and toxicity of nanoparticles using species of *Asteraceae* and their potential applications for human welfare. Constraints and future prospects for green synthesis of nanoparticles from members of the *Asteraceae* family are summarized.

## 1. Introduction

The *Asteraceae* (Aster, Compositae, Daisy) family represents around 1600 genera, of which around 80 are reported for nanoparticle synthesis. *Asteraceae* is a sophisticated and botanically immensely specialized family containing mostly herbs. They are found in the tropics and tropical and warm areas of South, Southeast, and East Asia, Africa, Madagascar, and Central South America. Several of the genera in this family, such as *Aster, Helianthus, Chrysanthemum*, and *Tagetes*, are ornamentals, most of which have therapeutic properties. Many members of this family are used in medicine. Some are commonly planted in the field for vegetable and nutritional purposes. The order Asterales is made up of a single family, the sunflower family, which is the biggest of all plant families, with approximately 20,000 species. The *Asteraceae* is not only a vast and widespread family, but also, as one might assume, a varied one. Evolution has been generated in several directions, and the main developmental pathways are characterized by combining related genera into tribes [1].

Around 300 *Asteraceae* species have been utilized for medicinal reasons. Phytochemical derivatives from *Artemisia annua*, for example, are used in the treatment of malaria. The endangered *Saussurea involucrata* had been used for anti-inflammatory, anti-tumor, and radical scavenging capabilities. Various *Asteraceae* family species such as *Lactuca sativa, Cichorium intybus, Smallanthus sonchifolius, Helianthus tuberosus*, etc., have been used as food crops. Seeds of *Asteraceae* species such as *Helianthus annuus* and *Carthamus tinctorius* have been used as cooking oil. *Chrysanthemum, Tanacetum*, and *Pulicaria* genera are proven to have insecticidal activities and are commercially significant members of the *Asteraceae* family [2]. Various ethnobotanical data on the traditional uses of *Asteraceae* species, particularly for blisters, breathing problems, miscarriage, pain, hypertension, runny nose, whooping cough, bowel problems, constipation, vomiting and diarrhea, metabolic syndrome, skin problems, infections, fracture, headache, cardiovascular disease, itchiness, anemia, menstruation illness, numbness, skin disorders, snake bites, sex issues, and dental illness is reported [3]. Plants present in this family are also used to cure different diseases such as tumors, sleeping sickness, indigestion, hepatotoxicity, epilepsy, etc. It also has antimicrobial, antioxidant, anti-proliferative, anti-inflammatory, and vasodilatory activities [4].

Recent studies reported that plants belonging to the *Asteraceae* family have an excellent ability to synthesize NPs in non-toxic ways and these NPs have numerous applications. Different NPs such as silver [5], gold [6], copper [7], iron oxide [8], and zinc oxide [9] are successfully synthesized from *Asteraceae* members. The biosynthesis of NPs using plant extract of *Asteraceae* members is simple, easily available, low cost, and eco-friendly [10,11]. Numerous studies offering experimental data on the biological impacts of *Asteraceae* species have grown in recent years. There is, however, no comprehensive systematic review that summarizes existing understanding. With extensive traditional knowledge and application of *Asteraceae* species, the current study attempted to compile all published research on their phytochemical extraction process for nanoparticle synthesis and pharmacological properties for the first time.

## 2. Plant-Based Green Synthesis of Nanoparticles

Green synthesis has acquired a lot of importance as a sustainable, economical, feasible, and environment-friendly synthesizing procedure for a variety of bio-inspired materials. Green synthesis helps in decreasing the harmful effects associated with nanoparticle synthesis by physical and chemical methods. Plant phytochemicals involved in green synthesis show greater reduction and stabilization properties. Biologically, the nanoparticle can be synthesized using bacteria, fungi, algae, and plants [12]. Among all these organisms, plants have a higher potential to produce the NPs because the synthesis of NPs using microorganisms is affected by culture contamination, lengthy procedures to produce adequate production of biomass, less control over NP size, and reuse of biomass for the subsequent nanoparticle synthesis. It is also difficult to maintain the microbial culture under aseptic conditions and the cost of isolation of microorganisms is not economically efficient [13]. At the same time, plant synthesis is more beneficial than the other methods due to its high stability, lack of contamination risk, easy preparation, and less time consumption [7,14]. Plants and their extracts act as natural chemicals because they contain phytochemicals such as flavonoids, terpenoids, phenols, polyphenols, amides, aldehydes, and saponins [15]. Reducing and capping the nature of phytochemicals and plant enzymes such as reductase help to reduce the NPs from metal ions. Plants eliminate the usage of expensive instruments, high-pressure, and hazardous chemicals [16].

## 3. *Asteraceae* Mediated Nanoparticle Synthesis: The Pursued Routes

**Figure 1:**
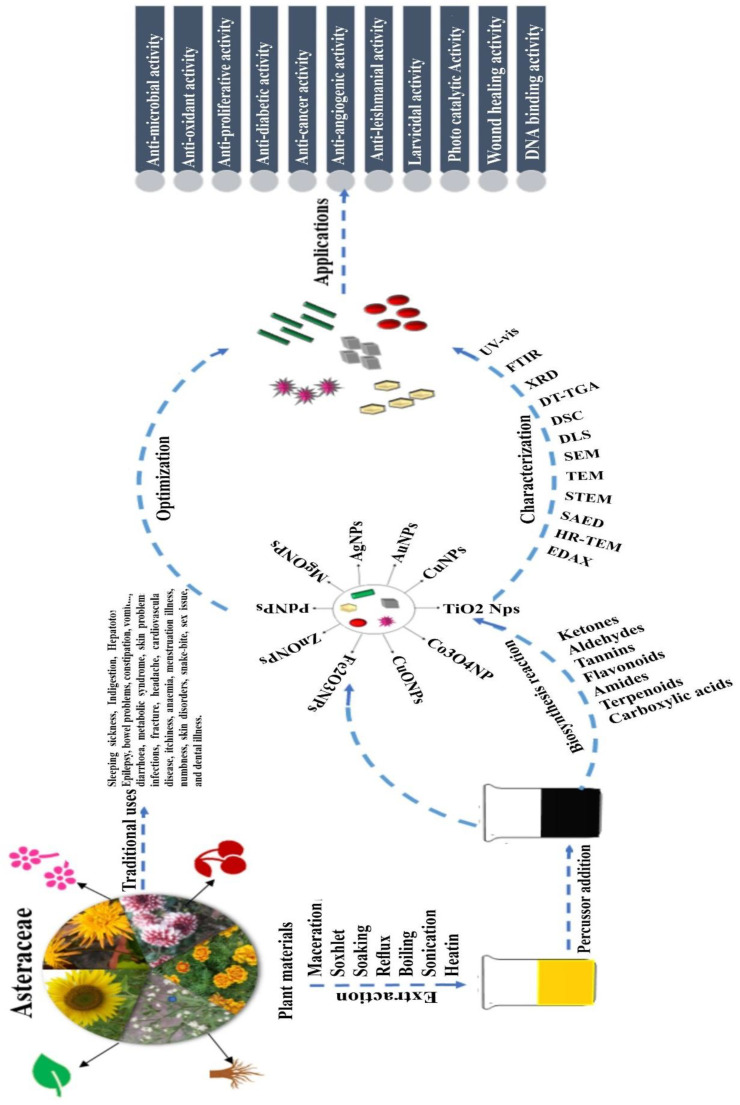
Flow chart showing the steps involved in the extraction, synthesis, optimization, and characterization of nanoparticles from the *Asteraceae* family and its applications.

Efficient synthesis, extraction, and identification of nanoparticles require optimization of parameters such as the plant material and solvents used, phytochemicals involved, factors affecting the synthesis, and characterization techniques utilized for identification (Figure 1, Table 1 and Table 2).

### 3.1. Plant Material Used

Roots, stems, leaves, fruits, flowers, and seeds of *Asteraceae* members were used for the synthesis of NPs. The parts which are used to synthesize the nanoparticle could be washed and heated using a solvent. Researchers reported that both fresh and dried samples could be used to synthesize NPs. Dried samples at room temperature go through the process of weighing and crushing. Using Whatman filter paper, solutions are filtered, and clear solutions are used for synthesis [107]. Several studies report NPs being successfully synthesized from leaves of *Acanthospermum hispidum*, [17], the stem of *Matricaria recutita* [65], roots of *Pechuelloeschea leubnitziae* [69], the flower of *Rhanterium epapposum* [72], and seeds of *Silybum marianum* [77], etc., of *Asteraceae* members.

### 3.2. Extraction Methods

Extraction is the first and crucial step in the production of NPs. It happens when the solvent is diffused into plant tissues and solubilized phytochemicals with similar polarity and also these phytochemicals in the plant extract function as biocatalysts. The plant extract can be extracted using different methods such as maceration, soaking, soxhlet, reflux, sonication, heating, and boiling methods. Maceration was used to prepare an aqueous extract of *Solidago canadensis* to synthesize gold NPs [103]. Leaf extracts of *Spilanthes calva* were prepared using a boiling method to synthesize silver NPs [146]. To synthesize silver NPs from the leaf extract of *Tragopogon collinus* both the soaking and boiling methods were used [88].

### 3.3. Solvents Used

The solvent-free synthesis is not achievable in nanoparticle synthesis, since solvents have a crucial role in transferring the heat, dissolving solids, purification, and isolation steps, and altering viscosity. In green synthesis, the solvent is used in large amounts when compared to other materials, so the choice of solvent is essential, and also the types of solvents used during extraction significantly affect the amount of reducing agents extracted. Benzene is proven to be the best solvent but cannot be used in the synthesis of NPs due to its carcinogenic nature. Predominantly distilled water in addition to organic solvents such as ethanol and methanol are used to prepare extracts for the NPs synthesis from *Asteraceae* members. Among all the solvents, water is the best choice for the synthesis of NPs as it is non-toxic, eco-friendly, non-flammable, and economically feasible [147]. *Tagetes erecta* aqueous extracts were used for nickel NPs synthesis [141]. However, methanol and ethanol extract is used to synthesize silver nanoparticles from *Tragopogon collinus* [88].

### 3.4. Phytochemicals Involved

**Figure 2:**
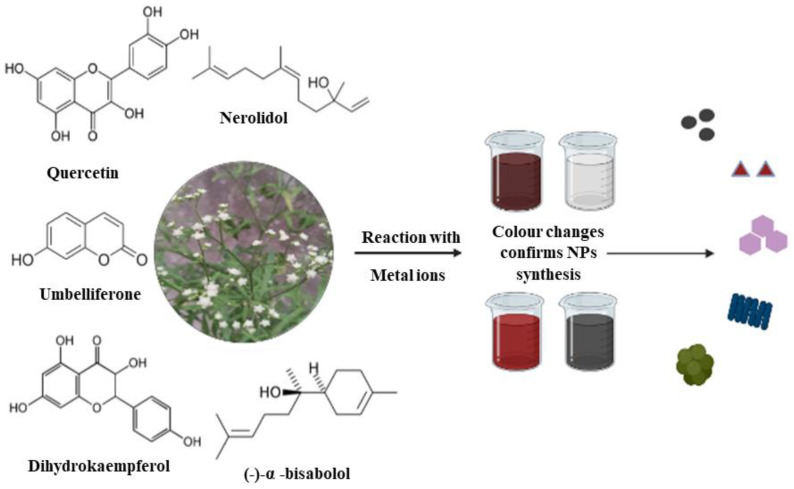
Summary of the role of phytochemicals present in *Asteraceae* family in reduction of metal ions to various nanostructured materials.

It is reported that the phytochemicals such as ketones, aldehydes, tannins, flavonoids, amides, terpenoids, and carboxylic acids in the plant are in charge of metal ion reduction (Figure 2). The compounds in the plant extract components, are capable of electron donation, causing metal ion reduction to NPs. Plant extract concentration also depends on the solvent used for the extraction process. Tannins help in the production of silver NPs by acting as reducing and capping agents and also water-soluble secondary metabolites such as proteins, amino acids, and phenol control the biosynthesis of silver NPs. Several studies report plant extract and phytochemical concentrations affecting the size, shape, and application of the nanoparticle [148]. *Tragopogon collinus* extract containing phenolic compounds play a prominent role in the production of NPs [88]. In UV spectrum analysis, the aqueous leaf extract of *Bidens pilosa, Galinsoga parviflora*, and *Conyza bonariensis* showed absorbance peaks at 288 nm, 267 nm, and 286 nm, respectively. These peaks confirmed the presence of sugars, polyphenols, and amino acids, helpful in Fe ions reduction to Fe NPs [128].

### 3.5. Nanoparticle Synthesis from Asteraceae Species

A large number of *Asteraceae* members have been utilized for the synthesis of various nanoparticles such as gold, silver, iron, copper, etc. For example, silver NPs synthesized from *Asteraceae* family members have significant catalytic action, atomic behavior, and biochemical reactivity due to large surface area. Recognition, reduction, limited nucleation, and growth help in the formation of silver NPs. In the recognition stage, the metal ions will be trapped on the surface protein of the plant extract by electrostatic interaction. Thereafter, proteins present in the extract reduce the Ag+ ions to Ag^0^ by changing the secondary structure of the protein. This causes Ag ions reduction and accumulation in nuclei. The linkage of protein and a large number of biomolecules in the solution may lead to isotropic growth and the production of stable NPs [149].

The NPs synthesized by the addition of silver solution to the extract via. the green method is detected by color change. The color change is an indicator of the Ag ions reduction to Ag NPs by the plant extract phytochemicals. Briefly, the phytochemical compounds such as polyphenols, terpenoids, etc., present in the extract of *Asteraceae* members, donate electrons to reduce metal ions and form zero-valent metal atoms. Eventually, the collision of metal atoms with these atoms in the mixture results in the formation of several atoms with a stable core. These atoms will perform as nucleation regions and will form clusters that will continue to grow till an active supply of atoms, results in NPs formation. The process is carried out by the reduction of metal ions into metal NPs [33]. Similarly, other metal ions are converted to respective metal NPs as plant extracts are capable of forming NPs by adding metal salt to the solution. The color change in metal NPs varies from each other for example, dark brown, wine red, reddish-brown, and white color for silver, gold, iron, and zinc oxide NPs, respectively.

#### 3.5.1. Factors Affecting the Synthesis of Asteraceae NPs

The green synthesis of metal nanoparticle formation is by metal ion reduction caused by phytochemical compounds present in *Asteraceae* members. Several factors affect the synthesis of NPs such as plant extract concentration, metal ions concentration, temperature, pH, and reaction time. These factors affect the size, shape, and distribution of NPs.

##### Temperature

During nanoparticle synthesis, temperature plays a crucial role in metal ion reduction to metal NPs. Normally, the reaction is carried out at room temperature, but it is also reported that some members of the *Asteraceae* family need a higher temperature to reduce the metal ions to metal NPs. Studies report silver NPs synthesized at room temperature (*Acanthospermum hispidum and Anthemis atropatana*), 40 °C (*Achillea biebersteinii*), 60 °C (*Centratherum anthelmminticum*), and 80 °C (*Arnicae anthodium*) [17,18,22,23,35]. UV–Vis spectroscopy explains that Ag NPs synthesized from leaves of *Arctium lappa* and *Eupatorium odartum* at 90 °C give an intense surface plasmon resonance (SPR) band and this intense SPR band indicates synthesis of NPs on a large scale [6,54]. At room temperature, gold NPs could be synthesized from *Centaurea behens* leaf extract when it is mixed with chloroauric acid [95]. Rectangular, cubic, and hexagonal-shaped Cu NPs can be synthesized using *Ageratum houstonianum* leaf extract at room temperature [13]. At 55 °C, a dark brown colored copper nanoparticle solution was formed from copper (II) nitrate trihydrate solution and aqueous leaf extract of *Calendula officinalis* [150]. In copper nanoparticle formation, when time increases the surface plasmon resonance decreases due to the oxidation of Cu NPs [151]. Metal oxide NPs such as iron oxide NPs and ZnO NPs were formed from *Artemisia* species and metal precursors at room temperature [114,127].

##### pH

pH is a significant parameter during the synthesis of NPs. NPs’ size, shape, and stability are affected by the reacting solutions’ nature, i.e., acidic and alkaline medium. Reports suggest large-sized NPs are formed in an acidic medium and small-sized NPs are formed in an alkaline medium. However, the conversion efficiency of NPs is high in an alkaline medium [152]. Studies on the pH effect on AgNPs’ formation using *Tithonia diversifolia* showed absorbance intensity increased gradually with an increase in pH range. However, in basic and neutral pH, the Ag NPs formation was very fast which was evident from the color change in the reaction mixture. However, at basic pH, there is a possibility of Ag ions precipitating as AgOH. Studies support pH 7 as the optimal pH to synthesize silver NPs [86]. pH was maintained at 5.4 to synthesize gold NPs from an aqueous extract of *Sphaeranthus indicus* and hydrogen tetrachloroaurate (II) trihydrate [153]. Different pH levels such as 9, 10, 11, and 12 were also used for the synthesis of zinc oxide NPs from *Tragopogon collinus* extract, and a broad peak was observed in pH 9 and a narrow peak showed in pH 12 solution. The broad peak could be due to the large particle size and the narrow peak due to the nanosized material. Therefore, pH 12 was concluded as best for zinc oxide NPs formation using *Tragopogon collinus* [125].

##### Reaction Time

Reaction time is a major factor in the synthesis of NPs. In *Asteraceae*-mediated nanoparticle synthesis, the formation of NPs takes place immediately after adding the metal precursor. Interestingly, the reduction and synthesis of silver NPs using *Bidens frondosa* extract were observed using UV–visible spectroscopic analysis. Silver nitrate solution addition to *B. frondosa* extract, Ag NPs synthesis started immediately and maximum production of AgNPs occurred at ambient temperature on 5 h of incubation [28]. The size, shape, and stability of the nanoparticle are also dependent upon the reaction time. The reaction time varies based on factors such as the concentration of metal ions, phytochemicals present, temperature, and pH of the plant extract [154]. Initially, the mixture of *Sphaeranthus indicus* extract and AuCl_4_ solution was light yellow color, it changed to a wine-red color after 30 min of stirring [153].

##### Metal Ion Concentration

Metal ion concentration depends upon which metal is being used to synthesize NPs. Studies reported that for silver nanoparticle synthesis, the frequently used concentration is 1 mM, and other concentrations (1, 2, 3, 5, 8, 10, 53, 100, and 200 mM) of metal NPs are synthesized [148]. Varying concentrations of zinc acetate dihydrate (0.05 to 0.25 M) were taken to synthesize zinc oxide NPs from the mixture of zinc acetate dihydrate and aqueous extract of *Tragopogon collinus*. Results showed that the absorption intensity was low at 0.2 M and high at 0.05 M. When metal ion concentration is increased beyond the threshold then gradually the nanoparticle synthesis will be decreased, and also higher concentration can lead to the agglomeration of the NPs [125]. Metal ions concentration also varies based on the presence of phytochemicals. The concentration of metal ions will also affect the size, shape, and uses of NPs [148].

##### Plant Extract Concentration

Concentration of plant extract depends upon the number of phytochemicals present in the plant. The concentration of phytochemicals varies among plants and within plant families. Studies revealed that 0.1 g to 10 g of plant parts were utilized to synthesize NPs [128,133]. An amount of 10 g of *Wedelia urticifolia* leaves was utilized to synthesize magnetic iron oxide nanorods. Similar studies revealed that 20 g dried powder of *Bidens pilosa*, *Galinsoga parviflora*, and *Conyza bonariensis* was utilized to synthesize iron NPs [128]. Investigation of the production of zinc oxide nanoparticles using *Tragopogon collinus*, different amounts of extracts (0.25, 0.5, 1, and 2 mL) were utilized. The result showed that 1 ml of the extract van reduce 50 mL of 0.01 M of zinc acetate dihydrate solution, for the synthesis of a large quantity of zinc oxide NPs. The optimum quantity or higher amount of the extract increases the intensity of the absorbance peak. The quantity of synthesized NPs increases when the phytochemical present in the extract is more. Hence, large quantities of extract increase the production of NPs with improved absorption intensity [125].

### 3.6. Separation of NPs

The centrifugation approach for purifying NPs is frequently used to remove residual components and byproducts. Apart from centrifugation, NPs can be separated using chromatography and electrophoresis techniques [155]. Appropriate separation and purification are critical for nanoparticle characterization and applications. As noted in the reviewed publications, the green synthesis produced a variety of forms and sizes, the majority of which were spherical and polydisperse, and was proven to be efficient for the creation of silver NPs. Green synthesis, compared to physical and chemical synthesis processes results in less controlled morphologies, which may be related to several reducing/capping phytochemicals, which cause multiple redox reaction rates and growth of the NPs [16].

### 3.7. Characterization

Characterization techniques are utilized for the determination of NPs’ form, shape, surface, and dispersion. UV–visible spectrophotometry (UV–Vis), dynamic light scattering (DLS), zeta potential, Fourier transform infrared spectroscopy (FT-IR), X-ray diffraction (XRD), differential scanning calorimetry (DSC) energy dispersive spectroscopy (EDS), selected area electron diffraction (SAED), thermogravimetric analysis (TGA), scanning electron microscopy (SEM), transmission electron microscopy (TEM), scanning transmission mode (STEM), etc., are some of the commonly used methods [156].

#### 3.7.1. UV–Visible Spectroscopy

UV–Vis is a relatively easier technique that permits rapid identification and characterization of NPs. Because of the interaction of light with movable surface electrons of NPs produces a significant absorbance band in the 400–500 nm range known as surface plasmon resonance (SPR) [157].

The UV–Vis absorbance peaks were observed in a range of 414 to 460 nm, 530 to 580 nm, 320 to 690 nm, 415 nm, 330 to 430 nm, 260 to 496 nm, 211 to 305 nm, 282 to 322 nm, 266 to 324 nm, and 250 to 320 nm for Ag NPs, Au NPs, Cu NPs, Pd NPs, ZnO NPs, Fe_2_O_3_ NPs, CuO NPs, TiO_2_ NPs, CO_3_O_4_ NPs, MgO NPs, respectively [8,17,94,107,112,134,137,140,142,143].

The copper NPs synthesized using *Achillea biebersteinii* leaf aqueous extract peaked at 577 nm [107]. Iron NPs synthesized using *Ageratum conyzoides* extracts were observed at 390 nm [8]. Biosynthesized titanium dioxide NPs by using *Echinacea purpurea* Herba extract that showed a peak at 280 nm [138]. Gold NPs synthesized using flower extract of *Carthamus tinctorius* showed a peak at 560 nm [158].

#### 3.7.2. Fourier Transforms Infrared Spectroscopy

The FT-IR reveals the surface properties of nanomaterials. This method aids in the identification of functional groups in both phytoconstituents and the resultant NPs. The FT-IR analysis of plant phytochemicals in free form or attached to NPs occasionally predicts minor band changes. There have been few studies on the use of pure phytochemical substances in the manufacture and use of NPs [45]. The list of nanoparticles synthesized from *Asteraceae* family, which characterized through FT-IR spectra described in Table 3.

The IR spectrum of silver Ag NPs synthesized from *Ageratum conyzoides* showed absorption bands at 3444.29, 2358.95, 1613.99, 1383.98, 1074.83, and 699.38 cm^−1^. The peak at 3440.29 cm^−1^ corresponds to amide N-H stretching. The peak observed at 2358.95 cm^−1^ may be due to the C-H stretching of the methylene group. The band at 1383.98 cm^−1^ corresponds to the presence of stretching vibrations of alcohol, esters, ethers, carboxylic acids, and amino acids [13]. The AuNPs peaks were observed at 415, 406, 394, 383, and 1629, which detect metal oxide bonds. The Cu NPs represent broad peaks at 3378 cm^−1^ and can be assigned to the phenolic compounds with OH bonds such as flavonoids, tannins, and glycoside derivatives. In addition, peaks at 1100 and 1700 cm^−1^ depict C-O and C=O stretching, respectively, of *Blumea balsamifera* leaf extracts [108]. The peaks 1264 and 1077 indicate the presence of C–O stretching of alcohols, carboxylic acids, and ester and ether groups in Pd NPs [112].

#### 3.7.3. X-ray Diffraction

XRD offers chemical information for both elemental and phase research. XRD is beneficial for measuring stress and analysis of texture, in addition to chemical characterization. XRD analysis requires crystalline samples, however, the technique can determine the degree of crystallinity in polymers. XRD has typically been used for bulk sample analysis. However, with the introduction of new optical techniques, the thin-film examination may now be performed [160].

The XRD pattern of CuNPs was synthesized from *Eclipta prostrata* leaves extract, showing the formation of a face-centered cubic (FCC) arrangement ranging from 23 to 57 nm, with an average size of 31±1.2 nm [109]. Peaks for AgNPs were observed at 38.2°, 44.1°, 64.1°, and 77.0° [18]. The 2θ values 38°, 44°, 64°, and 77° correspond to AuNPs [95]. The crystallinity of Pd NPs from *P. glutinosa* plant extract was confirmed by XRD analysis. Five distinct reflections in the diffractogram at 40.02° (111), 46.49° (200), 68.05° (220), 81.74° (311), and 86.24° (222) were observed, which predicts to FCC shape of palladium NPs [112]. The XRD pattern for ZnO NPs was 31.61°, 34.26°, 36.10°, 47.37°, 56.40°, 62.68°, and 67.72° [113]. The peaks appearing at 2 thetas of 19.86, 25.90, 26.11, 28.31, 29.82, 29.99, and 30.04 correspond to Fe_2_O_3_ NPs [8].

#### 3.7.4. Zeta Potential

The zeta potential indicates a nanoparticle’s charge concerning its surroundings. The zeta potential, however, is not a measurement of the molecule’s surface charge; rather, it is a measurement of the electric double layer formed by the surrounding ions in the solution. Zeta potential between 10 and +10 mV are essentially considered neutral, but zeta potential greater than +30 mV or less than 30 mV are strong cations and strong anions, respectively [161].

The zeta potential of synthesized AgNPs from *Centratherum anthalminticum* (L.) Kuntze was measured at −25.75 mV [35]. The zeta potential was observed at −31 mV suggesting the stability of AgNPs synthesized from *Artemisia marschalliana* [24]. The super-paramagnetic Fe_2_O_3_ NPs synthesized using the Stevia plant showed a magnitude of zeta potential observed at −41.1 mV [131]. The AuNPs synthesized by *Cichorium intybus* L. showed a zeta potential of −19.7 eV. Zeta potential measurement was performed to predict the surface charge and stability of NPs [96].

#### 3.7.5. Dynamic Light Scattering (DLS)

The sizing of NPs by DLS uses temporal variation of scattered light from suspended particles in Brownian motion to calculate their hydrodynamic size distribution [161,162]. It measures the hydrodynamic size, direct study of retention periods (also offers a hydrodynamic size), and differential refractometry or viscometry to assess macromolecular components’ molecular weight. [163]. The particle size of copper NPs synthesized by using *Ageratum houstonianum* Mill leaf extract was observed to be approx. 80 nm. The size of dispersed NPs was also confirmed by DLS analysis [13]. AuNPs synthesized by *Cichorium intybus* L. and *Elephantopus scaber* (Linn.) leaf extract showed the particle size 1.7–3.2 nm and 20–40 nm, respectively [96].

#### 3.7.6. Differential Scanning Calorimetry (DSC)

Melting characteristics and dependent melting temperature depression of synthesized nanomaterials are determined using DSC. The Gibbs–Thomson equation is utilized to study the size-dependent melting temperature property of alloy NPs, yielding a satisfactory prediction of melting temperature depression [163,164].

#### 3.7.7. Thermogravimetric Analysis (TGA)

In a controlled environment, the change in mass of a sample as a function of temperature and/or time is measured by TGA. A high-precision thermobalance is coupled to a pan/crucible holder within a temperature-controlled furnace to form the thermogravimetric analyzer used for TGA studies. The sample environment is controlled by a purge gas supplied into the furnace, such as nitrogen gas for an inert atmosphere or air/oxygen for an oxidizing atmosphere. Temperatures ranging from room temperature to 1000 °C are ideal for TGA studies [165].

After heating to 900 °C, the biosynthesized Ag/AgCl NPs using aqueous leaf extract of *Oedera genistifolia* preserved more than 70% of their original weight. Initial weight loss between 30–200 °C might be attributed to Ag/AgCl NPs moisture loss, and subsequent weight loss was detected. At 900 °C, the Ag/AgCl NPs preserved around 70% of their weight, indicating their resilience [67]. TGA provides the measure of biosynthesized IONPs from *Artemisia vulgaris* leaf extract weight as temperature varies over time. At temperatures below 200 °C, the mass of NPs varies by about 100%, indicating that the substance is related to water. At temperatures of up to 200 °C, IONPs begin to lose mass, indicating the breakdown of NPs coated biomolecule compounds [127].

#### 3.7.8. Selected Area Electron Diffraction (SAED)

SAED patterns were utilized to determine the typical morphological characteristics, framework, crystal structure, and chemical properties to identify the particles studied. For TiO_2_ rutile nano-size granules, a series of field examinations were carried out at various time frames and weather conditions to demonstrate the preliminary capability of these collecting and analysis methods [166]. SAED pattern for AgNPs synthesized using Matricaria recutita (Babunah) plant extract confirmed a spot pattern with XRD peak values <311>, <220>, and <111> planes [65].

#### 3.7.9. Scanning Electron Microscopy (SEM)

SEM pictures were captured in secondary electron mode (accelerating voltage of 10 kV) and processed with Image Tool software. The granule sizes were measured and compared to the Feret diameters. As metal sputtering sources, Pt/Pd and Cr targets (99.99% purity) were used, which create a configuration of distinct nanomaterials. A conducting sample of 6 m thick aluminum foil was used. Within the resolution range of the electron microscope utilized (1–3 nm), no NPs were found on its surface. Silica gel on chromatograms was used as a 2D nonconducting sample. Molecular sieves with well-developed 3D surface morphology were used as samples [167]. The investigation of NPs produced by magnet iron sputtering is also of interest to enhance experimental processes. SEM investigations of nonconducting materials are made more informative by the deposition of a metal onto a sample surface through magnetron sputtering [168].

The size and form of the Ag NPs produced from *Eclipta alba* leaf extract were measured, having a range of sizes from 310 to 400 nm [51]. The formation of AuNPs with *Gundelia tournefortii* L. possessed a spherical shape with an average diameter of 40–45 nm [102]. The Cu NPs size was confirmed to be 30–55 nm [108]. CuO NPs synthesized using *Anthemis nobilis* flowers show morphology-like rectangular structures ranging from 8–20 nm [135]. ZnO NPs synthesized using *Artemisia aucheri* are depicted as seabeds consisting of spherical and granular shapes in the range of 15–40 nm [169]. The nanoparticle sizes were observed in a range of 10 to 180 nm, 10 to 200 nm, 16 to 71 nm, 20 to 25 nm, 10 to 170 nm, 20 to 86 nm, 9 to 21 nm, 9 to 120 nm, 8 to 20 nm, 10 to 34 nm for AgNPs, AuNPs, CuNPs, Pd NPs, ZnO NPs, Fe_2_O_3_ NPs, CuO NPs, TiO_2_ NPs, CO_3_O_4_ NPs, MgO NPs, respectively [24,51,114,137,145,159].

#### 3.7.10. Transmission Electron Microscopy (TEM)

An electron beam imaging method for visualizing nanostructured samples that provide considerably higher resolution than light-based imaging techniques. Transmission electron microscopy is the best method for directly measuring nanoparticle size, grain boundaries, diameter, and morphological characteristics. The particle size range is wide, spanning from 1 nm to 5 nm. There is, however, a strong predilection for very tiny agglomeration. We divided them into four categories: FCC, icosahedral, decahedral, and twinned particles. It should be noted that our approach produces particles with an alkyl–thiol molecule passivating the surface [170].

The zinc oxide NPs synthesized using the *Artemisia pallens* plant extract showed a TEM result that shows a homogenous wurtzite structure [114]. The NiO NPs biosynthesized using *Tagetes erecta* L leaf extract revealed irregular forms of NPs [141]. The particle size ranges from 5 to 25 nm spherical particles for CuO NPs synthesized by *Acanthospermum hispidum* L. extract [134]. The spherical shape of AgNPs from *Erigeron bonariensis* with a particle size of 13 nm [53]. Gold NPs synthesized by *Solidago canadensis* L. extract showed a combination of single crystals and twinned particles [103]. The nanoparticle sizes were observed in a range of 10 to 100 nm, 10 to 50 nm, 20 to 50 nm, 5 to 50 nm, 20 to 70 nm, 5 to 60 nm, 12 to 50 nm, 5 to 50 nm, 8 to 20 nm, 5 to 25 nm for Ag, Au, Cu, Pd, ZnO, Fe_2_O_3_, CuO, TiO_2_, CO_3_O_4_, and MgO NPs, respectively [17,94,114,128,137,145,159].

#### 3.7.11. Scanning Transmission Mode (STEM)

The STEM can approach atomic resolution, enabling direct imaging of smaller dimensions previously unobservable using traditional electron microscopy techniques. Combining this model with a high-angle annular dark-field detector, where the contrast on the picture is generally proportional to Z (where n is near 2), it is possible to identify elements on materials just solely on their atomic weight difference. This direct interpretation is of particular importance in the catalysis sector since bimetallic NPs are utilized in a variety of processes, including CO oxidation, hydrocarbon hydrogenation, and vinyl acetate production, among others. Probes as small as one can now be made, single molecules can be photographed, and the structure and form of microscopic NPs as small as a few nanometers may be detected [171]. The silver NPs synthesized using *Ambrosia arborescens* were observed as spherical and dispersed in solution with an average particle size of 14 ± 6 nm [21].

## 4. Application of *Asteraceae*-Based Nanoparticles

**Figure 3:**
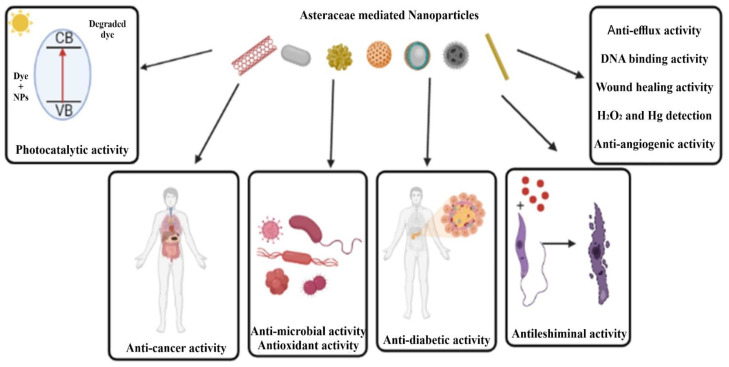
Role of *Asteraceae* mediated nanostructured materials in effluent treatment, drug delivery, antimicrobial, antioxidant, and other medical diagnoses.

Unlike the traditional application of plants from the *Asteraceae* family, green synthesized nanoparticles have shown highly significant biological responses. These may be attributed to the small size of these particles which can be targeted specifically for biological applications such as antimicrobial, anticancer, photocatalytic, etc. (Figure 1 and Figure 3, Table 1 and Table 2).

### 4.1. Antimicrobial Activity

Researchers have reported numerous antimicrobial activities by green synthesized NPs using *Asteraceae* members. NPs such as silver, copper, gold, iron oxide, zinc oxide, titanium oxide, nickel oxide, and copper oxides synthesized from different members of *Asteraceae* exhibited great antimicrobial activity. Most commonly, Ag NPs are synthesized from *Asteraceae* members as Ag is a safe non-toxic metal. Ag NPs have great potential because of their antimicrobial properties and were also used in the treatment of contaminated groundwater. Ag NPs are good antibiotics and preservatives [172], thus used in the food industry. The Ag NPs synthesized from *Carthamus tinctorius* showed antibacterial activity against toxic pathogens such as *Pseudomonas fluorescens* (ATCC 13867) and *Staphylococcus aureus* (ATCC 25923) in the food industry [33]. Ag NPs synthesized from leaf extract of *Eupatorium odaratum* exhibited a broad spectrum of antibacterial and antifungal potential against *Escherichia coli*, *Bacillus subtilis*, *S. aureus*, *Salmonella typhi*, and *Candida albicans*, respectively [54]. Leaves of *Tagetes erecta* were capable of synthesizing Ag NPs and showed antibacterial activity against *E. coli* (DH5-Alpha) and *Staphylococcus aureus* (ATCC9144™) [173]. Quasi-spherical shaped Ag NPs synthesized from *Acanthospermum hispidum* have antibacterial, antifungal, and antimycobacterial activity [17].

Similarly, *Tragopogon collinus* synthesized ZnO NPs exhibited antibacterial properties against *E. coli* (PTCC 1270) *and Staphylococcus aureus* (PTCC 1112) [125]. Synthesized *Cynara scolymus* ZnO NPs from leaf extract exhibit antimicrobial properties against *Staphylococcus aureus* (ATCC 25923), *Escherichia coli* (ATCC 25922) *Pseudomonas aeruginosa* (ATCC 27853) *Candida tropicalis* (IFM 46521), and *Candida albicans* (IFM 40009) [116]. *Parthenium hysterophorus*-mediated ZnO NPs (25 µL/mL) have good antifungal activity against *Aspergillus flavus* (MTCC-7589), and *Aspergillus niger* (MTCC-2587) [174]. *Ageratum conyzoides* can reduce iron metal to Fe NPs which have moderate antimicrobial activity against *Escherichia coli* (ATCC25922), *Bacillus subtilis*, *Staphylococcus aureus* (ATCC-25923), *Pseudomonas aeruginosa* (ATCC-27853), and *Candida albicans* (ATCC 90028) [8]. Recent studies also reported that CuO NPs synthesized from *Acanthospermum hispidum* showed antibacterial, and antimycobacterial activity against *Escherichia coli* (MTCC 443), *Pseudomonas aeruginosa* (MTCC 1688)*, Staphylococcus aureus* (MTCC 96) and *Streptococcus pyogenus* (MTCC 442) *and Mycobacterium tuberculosis* H37RV [134].

### 4.2. Antioxidant Activity

Antioxidants are substances that may remove, prevent, or delay cell damage caused by free radicals including reactive oxygen species (ROS), reactive nitrogen species (RNS), and other unstable molecules. DPPH (2,2-diphenyl-1-picryl-hydrazyl) assay is a commonly used method for the determination of antioxidant capacity [175]. Many researchers report that *Asteraceae*-mediated NPs have high antioxidant activity and can be used to treat diseases caused by oxidative stress and free radical-related disease. High antioxidant properties of *Asteraceae* members are accounted for by a large amount of phenolic and flavonoid content.

Studies report that synthesized Ag NPs from the leaf extract of *Ageratum conyzoides* has high antioxidant properties [13]. Ag NPs synthesized from *Calendula officinalis* are a good source of antioxidants because of their high antioxidant activity and can be used in the production of medicines and cosmetics [176]. Recent research proved that Au NPs synthesized from leaves of *Centaurea behen, Crassocephalum rubens, Gundelia tournefortii*, and seeds of *Cichorium intybus* can act as antioxidants [43,95,102]. Antioxidants were also produced from ZnO NPs synthesized from the flower of *Tagetes erecta* and seeds of *Zinnia elegans* [93,123]. Aqueous extract of *Silybum marianum* synthesized ZnO NPs showed antioxidant properties [122]. Cu NPs synthesized from *Blumea balsamifera*, and *Eclipta prostrata*, also showed antioxidant activities [108,109].

### 4.3. Anticancer Activity

NPs synthesized from the *Asteraceae* family have a higher potential for controlling the growth and multiplication of tumor cells. Ag NPs synthesized from *Artemisia marschalliana* and *A. turcomanica* exhibit anticancer activity in the human gastric cancer AGS cell line [24,25]. ZnO NPs from *Achillea millefolium* are highly stable and biocompatible. They showed cytotoxic activity on lung and colon cancer cells [177]. ZnO NPs from leaf extract of *Costus pictus* have cytotoxic activity against *Dalton lymphoma* ascites cells [9]. Au NPs from leaf extract of *Centaurea behen* showed anticancer activity against leukemia cell line [95]. ZnO NPs using leaf extract of *Cynara scolymus* were found to possess anti-proliferative activity against the human breast cancer cell line [116].

### 4.4. Antidiabetic Activity

Diabetes is a metabolic disorder that is developed due to glucose intolerance and hyperglycemia. It is also caused due to changes in food and lifestyle. A recent investigation reported that silver NPs synthesized from *Phagnalon niveum* methanol extract demonstrated antidiabetic activity by reducing the blood glucose level and also reduced the body weight of rats in 1 to 21 days [178]. Spherical shaped-CuO NPs synthesized from *Silybum marianum* seed extract displayed great enzymatic inhibitory activity against ureases, alpha-amylase, and lipases so it was concluded that they can act as antidiabetic agents [179]. ZnO NPs and Au NPs which are synthesized using *Dicoma anomala* and *Eclipta alba*, respectively, are good alternative sources for antidiabetic medicine [98,117].

### 4.5. Antileishmanial Activity

*Leishmania* is a parasitic protozoan that is a causative organism for oropharyngeal mucosa inflammation, cutaneous lesions, and visceral infections. Antileishmanial drugs are usually antimonial compounds, they are highly toxic. Pentavalent antimony drugs such as meglumine antimoniate and sodium stibogluconate are used in the initial treatment of leishmaniasis [180]. A recent study in green synthesis proved that zinc oxide NPs synthesized using *Silybum marianum* can replace toxic antimonial drugs to destroy *Leishmania tropica* (KMH23) which causes Leishmaniasis [122].

### 4.6. Anti-Angiogenic Activity

Angiogenesis has a major role in atherosclerosis, tumor growth, myocardial infarction, carcinogenesis, limb ischemia, and cardiac ischemia. Recent studies report Ag NPs synthesized from flower extract of *Achillea biebersteinii* can reduce angiogenesis. The anti-angiogenic activity of the silver nanoparticle was studied in the rat aortic ring model [18].

### 4.7. Photocatalytic Activity

Nanoparticles have been utilized for the degradation of various anionic, catatonic, and neutral dyes [181]. Dyes, commonly used in paper, plastic, food, cosmetics, leather, textile, and pharmaceutical industries and have proven to be harmful to both aquatic and human life due to their toxic, mutagenic, carcinogenic, and teratogenic effects. Research supports NPs synthesized from *Asteraceae* members as good catalysts to degrade the toxic dyes to non-toxic compounds. Ag NPs synthesized from leaf extract of *Ageratum conyzoides* showed photocatalytic degradation properties [13]. ZnO NPs formed from *Cynara scolymus* leaf extract were able to degrade 94.3% of methyl violet and 89.5% of malachite green dyes after 120 min of UV exposure [116]. A total of 83% of methylene blue was degraded by NiO NPs synthesized from leaf extract of *Ageratum conyzoides* [140]. Under solar light, TiO_2_ NPs synthesized from leaf extract of *Ageratina altissima* had the potential to degrade dyes such as crystal violet, methylene blue, alizarin red, and methyl orange [137]. FeO NPs synthesized from *Wedelia urticifolia* leaf extract and *Centaurea cyanus*, showed photocatalytic degradation activity and were used for the removal of toxic chemicals or dyes from the aquatic environment [129,133].

### 4.8. Other Activities

Nanoparticles synthesized from plants of the *Asteraceae* family revealed other applications such as anti-efflux activity, DNA binding, detection of mercury ions, cutaneous wound healing effect, electrochemical sensing activity, hydrogen peroxide detection, and tyrosinase inhibitory activity. Silver NPs synthesized from *Acroptilon repens* have been shown to have anti-efflux activity against clinical isolates such as *Acinetobacter bumanni* [19,129]. DNA binding and Hydrogen peroxide sensing properties have been found in *Agertum conyzoides* Ag NPs [13]. Ag NPs formed by the reduction of *Bidens frondosa* and Ag salt precursor showed tyrosinase inhibitory activity [28]. NiO NPs from the leaf extracts of *Tagetes erecta* have electrochemical sensing properties [141]. Au and Ag NPs synthesized from *Gundelia tournefortii* showed cutaneous wound healing activity [57,102] and Ag NPs synthesized from *Dahlia pinnata* were utilized for mercury ion detection [46].

## 5. Toxicity of *Asteraceae* Mediated Nanoparticles

NPs are highly toxic to the cells in comparison to large particles of the same chemicals. Studies concluded that the toxicity of NPs is inversely proportional to the size of the particles [182]. Several studies with the NPs synthesized from *Asteraceae* members have looked into how this toxicity can be used as an application to better suit the environment. Even with their toxicity to humans, low levels of NPs can still be used with an apt efficiency rate to reduce pollution as well as kill out several harmful living agents within our environment [183].

Ag NPs synthesized from the flower extracts of *Chrysanthemum indicum* have been proven to have lethal activity. These NPs can bring about the maximum mortality rate of *Anopheles stephensi* mosquitoes regardless of whether it is larvae or pupae [184].

The Cd NPs synthesized from *Tagetes* sp. showed a similar type of maximum mortality rate against *Aedes albopictus* at 72 h incubation while normal incubation only yielded 65 to 70% mortality. This showed that not only concentration but also incubation time can affect the toxicity that NPs have on particular mosquitos or other organisms. So, the ideal way to use NPs would be to use less concentration with high incubation time [185]. The leaf extract of *Ambrosia arborescens* and subsequent Ag NPs produced from the same plant extracts NPs had a dose-dependent toxic effect against *Aedes aegypti* larvae. However, no mortality rate was observed in the control groups [21]. Gold NPs from *Sphaeranthus indicus* extract did not have any particular toxic effect on the plant cells or aquatic invertebrates such as *Artemia nupulii* when tested with a particular similar dose-dependent concentration. However, it was shown to prompt the mitotic division of the root tip cells in *Allium cepa*, and also promoted the germination of pollen grains in *Gloriosa superba* [153].

While in the case of humans, toxicity is a bit different compared to the other fauna that has been characterized. NPs of size below 10 nm behave the same as gases, so they can easily enter through human tissue. After inhalation, NPs spread to the heart, lungs, spleen, liver, brain, and gastrointestinal tract and may disrupt the function of normal cells [182].

## 6. Constraints of *Asteraceae*-Mediated Nanoparticle Synthesis

*Asteraceae*-mediated nanoparticles have significant activities and applications, however, there are limitations in plant selection, synthesis process, nanoparticle quality assurances, and their applications. These limitations challenge the production of nanoparticles in large-scale and industrial production. Several plants in the *Asteraceae* family have been used to synthesize locally available nanoparticles. Yet, industrial production of *Asteraceae*-mediated nanoparticles is very hard to achieve due to the varying effects of climatic conditions, growing seasons, and large-scale cultivation of plants used for synthesis. Some of the very important concerns in the process of synthesis are long reaction time, pH, temperature, the use of chemicals, and excessive energy consumption. The challenge in the separation and purification of nanoparticles due to the interference of other phytochemicals in plants is another obstacle faced during the process of synthesis. The quality of obtained nanoparticles could be affected due to agglomeration, irregular shape, size, and low yield. Another limitation of *Asteraceae*-mediated nanoparticles is in their application, the efficiency of activities will be low, and time-consuming and large amounts of nanoparticles should be utilized for the same to achieve activities more efficiently.

## 7. Conclusions and Prospects

Extracts from plant parts such as leaves, roots, flowers, peels, stems, bark, and biological modifications were effectively employed for the synthesis of NPs under ambient circumstances under extremely moderate reaction conditions due to the clear potential efficacy and eco-friendliness of biogenic synthesis. UV–Vis, SEM, TEM, HR-TEM, STEM, SAED, XRD, EDAX, DT-TGA, FTIR, TGA, DSC, and DLS techniques, etc., were utilized for characterization. Biogenic NPs have shown remarkable anti-cancer, anti-diabetic, antibacterial, antifungal, and antioxidant properties. Under different temperature and pH conditions, the NPs remained stable for a longer amount of time. Phytochemicals in the plant extracts, such as polyphenols, polyphenolics, flavonoids, and other functional groups, different nanomaterial frameworks, and morphological characteristics were formed. *Asteraceae* is a large family with a vast number of beneficial plants. Silver, gold, copper, iron oxide, and zinc oxide NPs are successfully synthesized using *Asteraceae* members. NPs synthesized using *Asteraceae* members have huge applications such as antibacterial, antifungal, antiparasitic, antioxidant, photocatalytic degradation, and cytotoxic activities and thus need significant attention to be an important area of research in phyto-nanotechnology that provides new avenues towards the eco-friendly and economical synthesis of nanostructured materials. The mechanism involved in the synthesis of NPs, which is briefly through phytochemicals present in plants, aids in the reduction of metal NPs, but the exact mechanism remains unknown as to which phytochemicals play an important role in synthesis. It is said that collectively bioactive compounds aid in synthesis. It would be fascinating to learn which phytochemical molecule is responsible for green nanoparticle production.

## Figures and Tables

**Table 1:** Studies carried out to synthesize metal nanoparticles from the *Asteraceae* family and their biological applications.

Plant	Part Used	Solvent Used	Extraction Method	Phytochemicals	Characterization Techniques	SPR Peak (nm)	Nanoparticle Size (nm)	Activity	References
Silver NPs									
*Acanthospermum hispidum*	Leaf	DiW	Reflux	Saponins, coumarins, phenols, flavonoids, volatile oils, tannins, and sterols	UV–Vis, FE-SEM, EDX, TEM, FTIR, Particle size, and zeta potential	417	20–60	Antibacterial, antifungal, antimalarial, and antimycobacterial activity	[17]
*Achillea biebersteinii*	Flower	DDW	Boiling	Polysaccharides, polyphenols, and proteins	UV–Vis, TEM, zeta potential, and EDX	460	12 ± 2	Anti-angiogenesis activity	[18]
*Acroptilon repens*	Whole plant	DDW	Reflux	Caryophyllene oxide, α-copaene, β-caryophylene, and β-copaene-4-α-ol	UV–Vis, SEM, and TEM	420	38.89	Anti-efflux activity	[19]
*Ageratina adenophora*	Leaf	-	-	Carbohydrates, alkaloids, phenols, flavonoids, xanthoprotein, glycosides, tannins, steroids, and terpenoids	XRD, and FTIR	-	25	Antimicrobial activity	[20]
*Ageratum conyzoides*	Leaf	DDW	Boiling	Alkaloids, flavonoids, chromenes, benzofurans, and terpenoids	UV–Vis, FTIR, SEM, TEM, XRD, and EDX	443	14–48	DNA-binding, antioxidant, H_2_O_2_ sensing, and photocatalytic properties	[13]
*Ambrosia arborescens*	Leaf	DW	Stirring	Sesquiterpenic lactones, monoterpenes, terpenoids, and polyacetylenic resins	UV–Vis, FTIR, STEM, and SEM-EDX,	414	14 ± 6	Larvicidal activity	[21]
*Anthemis atropatana*	Aerial parts	Methanol	Boiling	Flavonoids, and phenolic compounds	UV–Vis, XRD, TEM, SEM, and FTIR	430	38.89	Antibacterial and cytotoxic activity	[21,22]
*Arctium lappa*	Whole plant	DW	Boiling	Phenolic acids, flavonoids, alkaloids, and terpenoids	UV–Vis, XRD, TEM, HRTEM, FTIR, EDX, TG, and DTA	435	21.3	Antimicrobial activity and catalyst for degradation of pollutants	[6]
*Arnicae anthodium*	Whole plant	DW	Boiling	Flavonoids. Triterpenes, sesquiterpene lactones and essential oils.	UV–Vis, FTIR, TXRF, and SEM-EDS	458	90–118	Antimicrobial activity	[23]
*Artemisia marschalliana*	Aerial parts	50% ethanol	Boiling	Phenolic acids and flavonoids	UV–Vis, XRD, FTIR, TEM, SEM, zeta potential, and EDS	430	5–50	Antioxidant, anticancer, and antibacterial activity	[24]
*Artemisia turcomanica*	Leaf	50% ethanol	Boiling	Phenolic acids, flavonoids, alkaloids and terpenoids	UV–Vis, TEM, SEM, XRD, and FTIR	430	22	Cytotoxic and anti-cancer activity	[25]
*Artemisia vulgaris*	Leaf	Methanol	Maceration	Phenolic acids, flavonoids, and alkaloids	UV–Vis, SEM, EDX, TEM, AFM, and FTIR	420	25	Antimicrobial, antioxidant, and antiproliferative activities	[26]
*Aspilia pluriseta*	Leaf	DW	Boiling	Flavonoids, phenols, alkaloids, and amino acids	UV–Vis, FTIR, SEM, DLS, TEM, and XRD	427	6	Antimicrobial and catalytic activity	[27]
*Bidens frondosa*	Whole plant	DW	Boiling	Terpenoids, phenolics and proteins	UV–Vis, FTIR, FESEM, and EDS	443	20–70	Tyrosinase activity	[28]
*Bidens pilosa*	Leaf, stem, and root	DW	Stirring	Terpenes, essential oils, tannins, polysaccharides, phenols, amino acids, ascorbic acid and organic acids	UV–Vis, SEM, TEM, EDX, and FTIR	410	17	Antimicrobial and anticancer activity	[29]
*Blumea eriantha*	Whole plant	Ethanol	Soxhlet	Phenols and flavonoids	UV–Vis, FTIR, SEM, XRD, and TEM	445	10	Antioxidant, antimicrobial, and cytotoxic activities	[30]
*Calendula officinalis*	Seed	DW	Boiling	Triterpenoids, flavonoids, coumarines, quinones, volatile oil, carotenoids, and amino acids	UV–Vis, TEM, XRD, and FTIR	440	05–10	-	[31]
*Carpesium cernuum*	Whole plant	Methanol	Reflux	Polyphenols	UV–Vis, and HR-TEM	430	13.0 ± 0.2	Antioxidant and anticancer activity	[32]
*Carthamus tinctorius*	Stem and Leaf	DW	Boiling	Flavonoids, polyphenols, proteins, sugars and saponins	HR-TEM, FTIR, and SEM	-	10	Antibacterial activity	[33]
*Centaurea virgata*	Aerial parts	N-hexane, chloroform, and methanol: water	Soxhlet	Flavonoids, phenolic acids, and terpenes	UV–Vis, FTIR, TEM, SEM, EDX, TGA XRD, and zeta potential	420	25–50	Antioxidant activity	[34]
*Centratherum anthalminticum*	Whole plant	DW	Heating	Phenolics and flavones	UV–Vis, XRD, SEM, FTIR, Particle size, DLS, and zeta potential	436	<50	Antimicrobial activity	[35]
*Chamaemelum nobile*	Whole plant	DW	Heating	Phenolics and flavones	UV–Vis, DLS, FTIR, XRD, and TEM	422	24.2 ± 3.1	Antibacterial activity	[36]
*Chromoleana odorata*	Leaf	-	-	-	UV–Vis, FTIR, XRD, SEM, FE-SEM, and EDX	428	20–25	Antibacterial activity and hydrogen peroxide detection	[37]
*Chrysanthemum indicum*	Flower	DW	Boiling	Flavonoids, terpenoids, and glycosides	UV–Vis, XRD, TEM, and EDX	435	37.71–71.99	Antibacterial and cytotoxic activity	[38]
*Chrysanthemum morifolium*	Flower	DW	Boiling	Flavonoids, caffeoylquinic acids, chlorogenic acid, phenolic acids	UV–Vis, FTIR, XRD, and TEM	430	20–50	Antibacterial activity	[39]
*Cichorium intybus*	Leaf	DDW	Boiling	Phenolic acids, triterpenoids, sterols, and hydroxycinnamic acid derivatives	XRD, FTIR, zeta potential, TEM, SEM, and EDS	-	17.17	Anticancer activity	[40]
*Cosmos caudatus*	Leaf	DW	Boiling	Phenolic acids, triterpenoids, and sterols	UV–Vis, XRD, FTIR, FESEM-EDX, and TEM	439	21.49 ± 7.43	-	[41]
*Cosmos sulphureus*	Leaf	DW	Boiling	Phenols, polyphenolic, and flavonoids	UV–Vis, Particle size, zeta potential, DLS, and SEM	430–440	55–80	Antimicrobial and antioxidant properties	[42]
*Crassocephalum rubens*	Leaf	DW	Boiling	Flavonoids, and polyphenols	UV–Vis, EDX, TEM, SEM, and FTIR	470	15–25	Antioxidant activity	[43]
*Cynara cardunculus*	Leaf	DW	Boiling	Polyphenols, flavonoids, and terpenoids	TEM, EDS, FTIR, and XPS	435	45	Antibacterial and electrochemical activity	[44]
*Cynara scolymus*	Leaf	DW	Heating	Alkaloids, polyphenols, flavonoid, and amino acid	UV–Vis, FTIR, SEM, EDX, and zeta sizer	434	98.47 ± 2.04	Anticancer activity	[45]
*Dahlia pinnata*	Leaf	DW	Boiling	Flavonoids, and phenolics	UV–Vis, XRD, TEM, and FTIR	460	15	Detection of Hg2+ ion	[46]
*Dicoma tomentosa*	Bark	DW	Boiling	Flavonoids, phenolic acids, and terpenes	UV–Vis	430–480	-	Antimicrobial activity	[47]
*Dittrichia viscosa*	Leaf	DW	Boiling	Flavonoids and polyphenols	UV–Vis, XRD, FTIR, and TEM	406	5–25	Bactericidal effects	[48]
*Echinacea purpurea*	Whole plant	DW	Heating	Caffeic acid derivatives, polysaccharides, alkaloids, alkylamides, and polyphenols	UV–Vis, XRD, SEM, and FTIR	481	68.24	Antioxidant activity	[49]
*Echinops* sp.	Root	DW	Heating	Carbohydrates, alkaloids, phenols, flavonoids, xanthoprotein, glycosides, tannins, steroids, and terpenoid	UV–Vis, UV-DRS, FTIR, XRD, SEM, EDXA, TEM, HRTEM, and SAED	454	33.86	Antimicrobial activity	[50]
*Eclipta alba*	Leaf	DW	Boiling	Phenols, flavonoids, and aldehydes	UV–Vis, DLS, FTIR, XRD, and SEM	433	310–400	Antimicrobial and cytotoxic activity	[51]
*Elephantopus scaber*	Leaf	DW	Boiling	Phenolics, amino acids, aliphatic, and aromatic hydroxyl groups	UV–Vis, NTA, TEM, XRD, and FTIR	435	50	Antioxidant activity	[52]
*Erigeron bonariensis*	Leaf	DW	Boiling	Terpenoids, flavonoids, and phenol derivatives	UV–Vis, SEM, EDX, TEM, XRD, AFM, and FTIR	422	13	Catalytic activity	[53]
*Eupatorium odoratum*	Leaf	DW	Boiling	Tannins, saponins, phytates, flavonoids, betacyanins, and alkaloids, steroids, terpenoids, phenols, quinones, and glycosides	UV–Vis, particle size, TEM, and PXRD	424	23.6	Antimicrobial and mosquito larvicidal activity	[54]
*Galinsoga formosa*	Leaf and Flower	DW	Boiling	Phenolics, amino acids, aliphatic, and aromatic hydroxyl groups	UV–Vis	350–400	-	Photocatalytic degradation activity	[55]
*Gazania rigens*	Whole plant	DW	Boiling	Flavonoids, polyphenols, proteins, sugars, and saponins	UV–Vis, XRD, EDX, and SEM	425–460	31.35	Antioxidant and photocatalytic degradation activity	[56]
*Gundelia tournefortii*	Leaves	DW	Stirring	Scopoletin, chlorogenic acids, terpinen-4-ol, linalool, zingiberene, caffeic acid, cymene, p-cymene, limonene, gallic acid, stigmasterol, aesculin, quercetin, and β-sitosterol.	UV–Vis, FE-SEM, TEM, XRD, and FTIR	419	16.5	Fungicidal, bactericidal, and cutaneous wound healing effects	[57]
*Gynura procumbens*	Leaves	DiW	Heating	Flavonoid and glycosides	UV–Vis, FTIR, TEM, and zeta potential	449–471	100	-	[58]
*Handelia trichophylla*	Flower	DiW	Stirring	-	UV–Vis, FESEM, EDX, TEM, FTIR, and XRD	448	20–50	Cytotoxic and antibacterial activity	[59]
*Helichrysum graveolens*	Shoot	DW		Flavonoid and other secondary metabolites	UV–Vis, FTIR, and TEM	439	11	Antimicrobial, anticancer, and photocatalytic degradation activity	[60]
*Jurinea dolomiaea*	Root	DW and methanol	Soaking	Phenols and flavonoids	UV–Vis, XRD, SEM, and FTIR	444	24.58	Antimicrobial activity	[61]
*Kleinia grandiflora*	Leaf	DiW	Boiling	-	UV–Vis, FTIR, XRD, SEM, TEM, and EDX	436–448	20–50	Antimicrobial, cytotoxicity, and photocatalytic degradation activity	[62]
*Lactuca sativa*	Leaf	Ultrapure water	Boiling	Polyphenols, flavonoids, sterols, triterpenes, triterpenoid saponins, beta-phenylethylamines, tetrahydroisoquinolines, reducing sugars such as glucose and fructose, amino acids, and proteins	UV–Vis, TEM, SEM, and FTIR	450	40–70	Antimicrobial activity	[63]
*Launaea taraxacifolia*	Leaf	DW	Heating	Alcohols, amides, and carbohydrates	UV–Vis, SEM, EDX, and TEM	440	9–15.5	Antibacterial activity	[64]
*Matricaria recutita*	Stem	DW and absolute ethanol	Boiling	Terpenoids, flavonoids, and coumarins	UV–Vis, SAED, HRTEM, and FTIR	445	11	Mercury ions sensor	[65]
*Mikania micrantha*	Leaf	DW	Boiling	Polyphenols, polyamides, and flavonoids	UV–Vis, FTIR, XRD, EDX, and TEM	425	5–20	Antibacterial activity	[66]
*Oedera genistifolia*	Leaf	DW	Heating	Phenolic, flavonoids, carbohydrates, terpenoids, and proteins	UV–Vis, FTIR, SEM, EDX, TEM, XRD, and TGA	400–500	34.2	Cytotoxic and antibacterial activity	[67]
*Parthenium hysterophorus*	Leaf	DW	Boiling	Alkaloids, glycoside, proteins, terpenoids, flavonoids, saponins, and tannins	UV–Vis, DLS, zeta potential, SEM, TEM, and FTIR	432	20–25	Anti-bacterial and antioxidant activity	[68]
*Pechuelloeschea leubnitziae*	Root	Hexane, dichloromethane, and methanol	Rotary evaporator	Saponins, anthraquinones, flavonoids, and polyphenols	UV–Vis, FTIR, XRD, EDX, and TEM	400	100	Anti-proliferative activity	[69]
*Pluchea sericea*	Leaf	DW	Heating	Flavonoids and phenolic compounds	UV–Vis, EDS, zeta potential, DLS, and EDS	487	59.2	Antibacterial activity	[70]
*Pulicaria glutinosa*	Whole plant	DiW	Reflux	Flavonoids and polyphenols	UV–Vis, XRD, TEM, EDX, and FTIR	422–459	40–60	-	[71]
*Rhanterium epapposum*	Flower	70% Methanol	Heating	-	UV–Vis, XRD, TEM, and FTIR	423	16.3	Antifungal and cytotoxic activities	[72]
*Sanvitalia procumbens*	Whole plant	DW	Heating	Flavonoids, phenolic groups, organic acids, and proteins	UV–Vis, FTIR, XRD, EDX, and SEM	438	46	Photocatalytic degradation activity	[73]
*Saussurea costus*	Root	-	-	-	UV–Vis, SEM, TEM, EDX, and FTIR	420	5–15	Photocatalytic degradation activity	[74]
*Scorzonera calyculata*	Aerial part	Ethanol and water	Stirring	Phenolic acid, flavonoids, alkaloids, and terpenoids	UV–Vis, TEM, SEM, FTIR, and XRD	420	25.28	Antibacterial, anticancer, and antioxidant activity	[75]
*Seripheidium quettense*	Aerial part	DW	Boiling	Phenols and flavonoids	UV–Vis, FTIR, XRD, SEM, TEM, and EDX	428	48.40–55.35	Antibacterial, antifungal, and cytotoxic activity	[76]
*Silybum marianum*	Seed	DW	Boiling	Proteins, polysaccharides, and flavonoids	UV–Vis, XRD, and TEM	425	1–25	-	[77]
*Solidago altissima*	Leaf	Millipore water	Boiling	-	UV–Vis, FTIR, EDS, SEM, TEM, and XRD	462	111	Antibacterial and photocatalytic activity	[78]
*Solidago canadensis*	Leaf	DW	Boiling	-	UV–Vis, and TEM	-	180.6	Cytotoxic activity	[79]
*Spilanthes calva*	Leaf	DW	Boiling	-	UV–Vis, SEM, EDAX, and FTIR	448.5	5–50	-	[80]
*Stevia rebaudiana*	Leaf	70% Ethanol	Heating	Flavonoids, phenolic acids, fatty acids, proteins, and vitamins	UV–Vis, and SEM	450	16–25	-	[81]
*Synedrella nodiflora*	Leaf	-	-	-	UV–Vis, FTIR, and XRD	460	-	Antimicrobial activity	[82]
*Tagetes erecta*	Flower	DiW	Boiling	-	UV–Vis, FTIR, XRD, SEM, and EDAX	420	24–49	Photocatalytic degradation activity	[83]
*Tanacetum vulgare*	Fruit	Ultrapure water	Boiling	-	UV–Vis, TEM, XRD, EDX, and FTIR	452	10–40	-	[84]
*Taraxacum officinale*	Leaf	Milli-Q water	Boiling	Flavonoid and phenolics acids (caffeic acid, and chlorogenic acid)	UV–Vis, XRD, FTIR, and HR-TEM	435	15	Antimicrobial, antioxidant, and anticancer activity	[85]
*Tithonia diversifolia*	Leaf	DW	Boiling	Proteins, polysaccharides, and terpenoids	UV–Vis, TEM, EDX, TG-DTA, and FT-IR	435	25	Antimicrobial activity	[86]
*Tragopogon buphthalmoides*	Whole plant	DW	Boiling	-	UV–Vis, XRD, FESEM, TEM and FTIR	420	-	Photocatalytic degradation activity	[87]
*Tragopogon collinus*	Leaf	Ethanol and methanol	Soaking and boiling	-	UV–Vis, TEM, XRD, and FT-IR	400	7	Antibacterial activity	[88]
*Verbesina encelioides*	Leaf and stem	DiW	Boiling	Sesquiterpenes, flavonoids, galegine, triterpenoids friedelin, epifriedelin, lupeol, a-, b-amyrin, stigmasterol, botulin, and bsitosterol	UV–Vis, FTIR, SEM, and XRD	430	54.6	Antimicrobial activity	[89]
*Vernonia amygdalina*	Leaf	Ethanol, 50% ethanol, DiW	Sonication	-	SEM, TEM, EDX, and FTIR	-	41.555 ± 2.488	Anticancer activity	[90]
*Vernonia cinerea*	Leaf	DDW	Boiling	-	UV–Vis, TEM, XRD, and FTIR	430	5–50	Antibacterial activity	[91]
*Wedelia chinensis*	Leaf	Milli-Q water	Boiling	Flavonoids and polyphenols	UV–Vis, TEM, EDX, XRD, XPS, and FTIR	408	31.68	Antioxidant, antibacterial and cytotoxic activity	[92]
*Xanthium strumarium*	Leaf	DiW	Boiling	Alkaloids, flavonoids, triterpenoids, terpenoids, tannin, saponin, quinone, protein, and sugars	HRTEM, SAED, FESEM, EDX, XRD, AFM, and FTIR	436	-	Antibacterial and antileishmanial activity	[15]
*Zinnia elegans*	Seed	-	-	-	UV–Vis, and DLS	439	79.5	Antioxidant activity	[93]
Gold NPs									
*Arctium lappa*	Whole plant	DDW	Heating	-	UV, SEM, TEM, FTIR, and AFM	580	10–40	Cytotoxic activity	[94]
*Centaurea behen*	Leaf	DiW	Boiling	Flavonoids, alkaloids, sesquiterpene lactones, lignans, chlorogenic, caffeic, ferulic, p-coumaric acids, isoquercitrin, and coumarin	UV–Vis, FTIR, XRD, EDX, and TEM	538	50	Antioxidant and anticancer activity	[95]
*Cichorium intybus*	Seed	DDW	Reflux	Alkaloids, inulin, sesquiterpene lactones, coumarins, vitamins, chlorophyll pigments, unsaturated sterols, flavonoids, saponins, tannins, and polyphenols.	UV–Vis, DLS, TEM, zeta potential, XRD, and FTIR	540	10–30	Antiproliferative, antioxidant, and photocatalytic activities	[96]
*Crassocephalum rubens*	Leaf	DW	Boiling	Flavonoids and polyphenols	UV–Vis, TEM, SEM, and FTIR	540	15–25	Antioxidant activity	[43]
*Echinacea angustifolia*	Flower	DW	Heating and stirring	Flavonoids, phenolics, flavones, and terpenoid	UV–Vis, FTIR and SEM	560	80–120	Antibacterial activity	[97]
*Eclipta alba*	Whole plant	Methanol	Soxhlet	-	UV–Vis, XRD, FTIR, DLS, TEM, SEM, and AFM	536	26	Antibacterial, antidiabetic, and anti-apoptotic activity	[98]
*Elephantopus scaber*	Leaf	-	-	-	UV–Vis, FTIR, SEM, and TEM	540	20–40	Anticancer activity	[99]
*Erigeron annuus*	Flower	-	-	-	UV–Vis, HR-TEM, XRD, EDS, FTIR and zeta potential	537	20–100	Catalytic activity	[100]
*Eupatorium odoratum*	Leaf	DiW	Heating	-	UV–Vis, DLS, FTIR, and TEM	528	10–20	Catalytic activity	[101]
*Gundelia tournefortii*	Leaf	DW	Soxhlet	-	UV–Vis, FTIR, FESEM, and EDS	528	40–45	Cytotoxicity, antioxidant, antibacterial, antifungal, and cutaneous wound healing activity	[102]
*Rhanterium epapposum*	Flower	Methanol	Heating	-	UV–Vis, XRD, TEM, and FTIR	525	17.9	Antifungal and cytotoxic activities	[72]
*Solidago canadensis*	Leaf	DDW	Maceration	Flavonoids, phenolic acids, glucosides, polysaccharides, diterpenes, triterpenoid saponosides, saponins, tannins, and essential oils	UV–Vis, ATR-FTIR, XRD, TEM, EDX, SAED, and SEM	530	8–200	-	[103]
*Stevia rebaudiana*	Leaf	Methanol	Soxhlet	-	UV–Vis, FTIR, XRD, SEM, and TEM	500–550	17	-	[104]
*Taraxacum officinale*	Whole plant	DW	Heating	-	UV–Vis, SEM, TEM, and XRD	500–600	15	-	[105]
*Xanthium strumarium*	Leaf	DiW	Heating	-	UV–Vis, FTIR, XRD, SEM, and TEM	-	9.60–11.70	Antibacterial and antifungal activity	[106]
Copper NPs									
*Achillea biebersteinii*	Leaf	DW	Stirring	Phenolics, anthraquinone, alkaloids, steroids, flavonoids, saponin, and tannin	UV–Vis, FTIR, EDS, TEM, and FESEM	577	16.8	Cytotoxic activity	[107]
*Ageratum houstonianum*	Leaf	DDW	Heating	Flavonoids, alkaloids, tannins, terpenes, steroid, and saponins,	UV–Vis, XRD, SEM, FTIR, TEM, and particle size analyzer	-	~80	Photocatalytic and antibacterial activity	[13]
*Blumea balsamifera*	Leaf	Ethyl acetate, n-hexane, and acetate	Rotary evaporator	Flavonoids and terpenoids	UV–Vis, SEM, and EDX	540	30–55	Antioxidant and cytotoxicity activity	[108]
*Eclipta prostrata*	Leaf	DW	Boiling	Thiophene-derivatives, steroids, triterpenes, flavonoids, polyacetylenes, polypeptides, and coumestans	UV–Vis, XRD, SEM, FTIR, EDX and HRTEM	695	31 ± 1.2	Antioxidant and cytotoxicity activity	[109]
*Pluchea sericea*	Leaf	DDW	Boiling	Phenols, flavonoids, and proteins	FTIR, EDS, and SEM	-	68.1	Insecticide activity	[110]
*Tridax procumbens*	Leaf	DW	Boiling	Alkaloid, carbohydrates, phenols, flavonoids, protein, amino acids, and phytosterol	UV–Vis, FTIR, SEM and XRD	320	71	Antioxidant, antibacterial, photocatalytic degradation activity	[111]
Palladium NPs									
*Pulicaria glutinosa*	Whole plant	DiW	Reflux	Polyphenolic and flavonoidic groups	UV–Vis, XRD, TEM, EDX, and FTIR	415	20–25	Catalytic activity	[112]

Note: UV–Vis: UV–Visible spectrophotometry; SEM: scanning electron microscopy; TEM: transmission electron microscopy; HRTEM: high resolution transmission electron microscopy; STEM: scanning transmission electron microscopy; SAED: selected area electron diffraction; XRD: X-ray crystallography; EDAX: energy dispersive X-ray analysis; DT-TGA: differential thermo gravimetric analysis; FTIR: Fourier transform infrared spectroscopy; TGA: thermal gravimetric analysis; DSC: differential scanning calorimetry; DTA: differential thermal analysis; TXRF: total reflection X-ray fluorescence; PPMS: physical property measurement system; VSM: vibrating sample temperature; EDXRF: energy dispersive X-ray fluorescences; BET: Brunau–Emmet–Teller analysis; XPS: X-ray photoelectron spectroscopy; AFM: atomic force microscopy; DLS: dynamic light scattering method; nm: nanometer; DW: distilled water; DDW: double distilled water; DiW: deionized water; SPR: surface plasmon resonance—: not available.

**Table 2:** Studies carried out to synthesize metal oxide nanoparticles from *Asteraceae* family and their biological applications.

Plant	Part Used	Solvent Used	Extraction Method	Phytochemicals	Characterization Techniques	SPR Peak (nm)	Nanoparticle Size (nm)	Activity	References
Zinc oxide NPs									
*Arctium lappa*	Whole plant	DDW	Heating and stirring	Polyacetylenes, arctinol, arctinal, arctinon, guaiane lactones, lignans, flavonoids, phenolic acids, inulin phytosterols, essential oil potassium, magnesium, and calcium salts, sesquiterpene bitter	UV, SEM, TEM, FTIR, and AFM	350	10 to 40	Cytotoxic activity	[94]
*Artemisia annua*	Whole plant	-	Heating and stirring	-	UV, FTIR, XRD, and TEM	330	20	Cytotoxic activity	[113]
*Artemisia pallens*	Whole plant	DDW	Distillation	-	UV, FTIR, XRD, SEM, and TEM	370	50–100	Antimicrobial activity	[114]
*Artemisia scoparia*	Whole plant	-	-	-	UV, FT-IR, XRD, TEM, FESEM, EDX, DLS, and zeta potential	370	9.00 ± 4.00	Anticancer activity	[115]
*Cynara scolymus*	Leaf	DW	Boiling	Phenolics acids, bitter sesquiterpenes lactones, and flavonoids	UV, FTIR, SEM, TEM, EDXA, and XRD	371	65	Antimicrobial, antiproliferative, and photocatalytic activity	[116]
*Dicoma anomala*	-	-	-	Alkaloids, flavonoids, tannins, and saponins	UV–Vis, TEM, FTIR, EDS, and XRD	386	-	Antidiabetic activity	[117]
*Dittrichia graveolens*	Whole plant	-	-	-	UV–Vis, FTIR, and FESEM	285–320	100	-	[118]
*Echinacea angustifolia*	Flower	DW	Heating and stirring	Flavonoids, phenolics, flavones, and terpenoids	UV–Vis, FTIR, and SEM	368	90–170	Antibacterial activity	[97]
*Lactuca sativa*	Whole plant	-	-	-	SEM, zeta potential, and DLS	-	90	-	[119]
*Parthenium hysterophorus*	Leaf	DDW	Heating	-	UV–Vis, SEM, TEM, and SEM-EDX,	400	16–45	Antibacterial activity	[120]
*Saussurea lappa*	Root	Methanol	Soaking	-	UV–Vis, FTIR, XRD, FESEM, and EDX	430	26 ± 1	Cytotoxic, antibacterial, and antifungal activities	[121]
*Silybum marianum*	Whole plant	DW	Heating and stirring	Polyphenols and flavonoids	UV–Vis, FTIR, XRD, HRSEM, and HRTEM	374	25	Antibacterial, antifungal, cytotoxicity, antileishmanial, antioxidant, and enzyme inhibition activity.	[122]
*Tagetes erecta*	Flower	-	-	Alkaloids, flavonoids, carbohydrates, amino acids, tannins, and proteins	UV, XRD, and SEM	364.15	30–50	Antioxidant, antimicrobial, and cytotoxic activities	[123]
*Tithonia diversifolia*	Leaf	DDW	Heating and stirring	Flavonoid, tannin, glycoside, alkaloids, saponin, steroids, and phenol.	UV–Vis, FTIR, XRD, SEM, EDX, and TEM	385	9.83–28.85	Dye degradation activity	[124]
*Tragopogon collinus*	Leaf	Ethanol	Boiling	Phenols	UV–Vis, TEM, XRD, and FT-IR	369	21	Antibacterial activity	[125]
*Vernonia amygdalina*	Leaf	Ethanol	Heating and stirring	-	UV–Vis, SEM, FTIR, XRD, and EDX	347	9.5	-	[126]
*Zinnia elegans*	Seed	-	-	-	UV–Vis, and DLS	350	82.6	Antioxidant activity	[93]
Iron Oxide NPs									
*Ageratum conyzoides*	Whole	DW	Boiling	Phenols and flavonoids	UV–Vis, FTIR, XRD, SEM, and SEM-EDX	390	85.9	Antimicrobial and photocatalytic activity	[8]
*Artemisia vulgaris*	Leaf	DiW	Heating	-	TEM, PSA, XRD, FTIR, VSM, and TGA	-	30	Photocatalytic degradation activity	[127]
*Bidens pilosa*	Leaf	DW	Heating	Phenols and flavonoids	UV–Vis, FTIR, EDXRF, XRD, and SEM	288	-	Photocatalytic degradation activity	[128]
*Centaurea cyanus*	Whole	DDW	Heating	Polyphenols, phenols, and flavonoids	XRD, BET, FTIR, and FE-SEM	-	24	Photocatalytic degradation activity	[129]
*Galinsoga parviflora*	Leaf	DW	Heating	Phenols and flavonoids	UV–Vis, FTIR, EDXRF, XRD, and SEM	267	-	Photocatalytic degradation activity	[128]
*Mikania mikrantha*	Leaf	DDW	Boiling	-	UV–Vis, XRD, SEM, TEM, and FTIR	-	20.27	Antimicrobial activity	[130]
*Stevia*	Whole	DiW	-	-	XRD, FESEM, HRTEM, TGA, XPS, VSM, and zeta potential	-	20	Antioxidant activity	[131]
*Vernonia amygdalina*	Leaf	DiW	Boiling	-	UV, FTIR, XRD, and SEM	396	-	-	[132]
*Wedelia urticifolia*	Leaf	DDW	Heating	-	UV, FTIR, XRD, TEM, and PPMS.	320	70	Photocatalytic degradation activity	[133]
Copper Oxide NPs									
*Acanthospermum hispidum*	Leaf	DiW	Reflux	Coumarins, tannins, saponins, phenols, flavonoids, sterols, and volatile oils	FESEM, EDX, TEM, XRD, and FTIR	-	9–21	Antimicrobial, antimalarial and antimycobacterial activity	[134]
*Anthemis nobilis*	Flower	DDW	Reflux	Luteolin-7-O-glucoside, apigenin-7-O-apioglucoside, and apigenin-7-O-glucoside.	UV–Vis, SEM, EDS, XRD, and FTIR	250	-	Catalytic activity	[135]
*Eupatorium odoratum*	Leaf	DW	Boiling	Flavonoids, phenolic compounds, and triterpenoids	UV–Vis, FTIR, XRD, SEM, TEM, and EDAX	211 and 305	-	Antibacterial activity	[136]
Titanium oxide NPs									
*Ageratina altissima*	Leaf	DDW	Boiling	-	UV–Vis, FTIR, XRD, and FESEM	332	60–100	Photocatalytic degradation activity	[137]
*Echinacea purpurea*	Whole plant	DDW	Boiling	Alkamides, cichroic acid, and polysaccharides	UV–Vis, SEM, TXRF, and FTIR	280	120	-	[138]
*Sonchus asper*	Leaf	DW	Soxhlet	-	XRD, FTIR, and FESEM	-	9–15	Antimicrobial activity	[139]
Nickel oxide NPs									
*Ageratum conyzoides*	Leaf	Methanol	Maceration	Alkaloids, tannins, phenols, saponin, and flavonoids	UV–Vis, FTIR, particle size, XRD, and TEM	324	8–15	Photocatalytic activity	[140]
*Tagetes erecta*	Leaf	DDW	Boiling	Flavonoids and phenols	XRD, SEM-EDX, TEM, and XPS	266–285	18.2	Photocatalytic, electrochemical sensing, and antibacterial activity	[141]
Cobalt Oxide NPs									
*Taraxacum Officinale*	Leaf	DiW	Soaking	Flavonoids and phenols	UV–Vis, FTIR, SEM, and TEM	319	50–100	Catalytic activity	[142]
Magnesium oxide NPs									
*Artemisia abrotanum*	Whole plant	DW	Magnetic stirring	Polyphenols, flavonoids (aglycones and glycosylates), and hydroxycinnamic derivatives	UV–Vis, FTIR, XRD, SEM, and TEM	300	10	Antioxidant and photocatalytic activity	[143]
*Chromolaena odorata*	Leaf	DiW	Water bath	Alkaloids, flavonoids, tannins, and other phenolic compounds	UV–Vis, FTIR, SEM, EDX, TEM, XRD, TGA, and DTA	270	12.3	Antimicrobial and catalytic activity	[144]
*Saussurea costus*	Root	Methanol	Reflux	Sesquiterpenes, alkaloid, triterpenes, lignans, and tannins	UV–Vis, FTIR, XRD, SEM, zeta potential, and DLS	250 and 320	34	Antimicrobial, anticancer, and photocatalytic activity	[145]

Note: UV–Vis: UV–Visible spectrophotometry; SEM: scanning electron microscopy; TEM: transmission electron microscopy; HRTEM: high resolution transmission electron microscopy; STEM: scanning transmission electron microscopy; SAED: selected area electron diffraction; XRD:X-ray crystallography; EDAX: energy dispersive X-ray analysis; DT-TGA: differential thermo gravimetric analysis; FTIR: Fourier transform infrared spectroscopy; TGA: thermal gravimetric analysis; DSC: differential scanning calorimetry; DTA: differential thermal analysis; TXRF: total reflection X-ray fluorescence; PPMS: physical property measurement system; VSM: vibrating sample temperature; EDXRF: energy dispersive X-ray fluorescence; BET: Brunau–Emmet–Teller analysis; XPS: X-ray photoelectron spectroscopy; AFM: atomic force microscopy; DLS: dynamic light scattering method; nm: nanometer; DW: distilled water; DDW: double distilled water; DiW: deionized water; SPR: surface plasmon resonance—: not available.

**Table 3:** Fourier transform infrared (FT-IR) spectra of the nanoparticles synthesized from *Asteraceae* family.

Plant Name	FTIR Absorption Bands (cm^−1^)		Possible Functional Group	References
	Plant Extract	NPs		
Silver NPs				
*Acanthospermum hispidum*	3786	-	-OH	[17]
	2964	-	C-H	
	1706	-	C=O	
	1601	-	C=C	
	1016	-	C-O	
*Ageratum conyzoides*	-	3440.29	N-H stretching	[13]
	-	2358.95	C-H	
	1383.98	-	Alcohol, ethers, esters, carboxylic acids, and amino acids	
	1613.99	-	C=O	
	1074.83	-	C-OH	
*Ambrosia arborescens*	-	1570	C=C	[21]
	-	1050	CO	
	1337.47	-	O-H	
	3280	-	OH	
	-	-		
*Anthemis atropatana*	-	1014	C-O	[22]
	-	1048	C-O stretching	
	3344	1595	N-H bending	
	-	2368	Cyanide	
*Arctium lappa*	596	632	C-Cl stretching	[6]
	1033	1036	C-N	
	1336	1384	N-H	
	2870	2853	C-H stretching	
	3375	3375	O-H stretching	
*Arnicae anthodium*	3284	-	-OH stretching	[23]
	2853	-	-C-H stretching	
	1735	-	C=C	
	1622	-	C=O	
	1370	-	-C-O	
	1027	-	-C-O-C	
	-	430, 395	-OH	
*Artemisia marschalliana*	3463	-	O-H	[24]
	3510	-	Protein binding	
	2962, 2823	-	C-H	
	1624	-	C-O	
	-	1398	C-N	
	1049	1038	C-O-C	
*Artemisia turcomanica*	13,429	3429–3473	O-H	[25]
	3029	-	C-H	
	2929	-	Aliphatic group	
	1635	-	C=O	
	1459	-	CH2	
	1273	-	C-O-C phenolic stretching	
	1064, 1119, 1168	-	C-O-C	
	1201	-	C-O-C stretching	
	1000	-	C=C-H	
	-	1635–1624	Carbonyl amide group	
	-	1382	N=O	
*Artemisia vulgaris*	3419, 3151	-	O=H	[26]
	1619	-	-C=O	
	1400	-	-C-N	
	1069	-	-C-O	
*Carthamus tinctorius*	3293	-	-OH	[33]
	2932	-	C-H	
	1725	-	C=O	
	1599	1533	C=C	
	1414	-	C=C aromatic	
	1053	-	C-O	
	860	-	C-H	
	818	-	#ERROR!	
	776	323	N-H	
*Chrysanthemum indicum*	3293	-	–OH	[38]
	2932	-	C–H	
	1725	-	C=O	
	1053	-	C=C,C–O–H	
	1599	-	C=C	
	-	1288 to 1299	Ag	
*Chrysanthemum morifolium*	1406	-	C=C group	[39]
	1078	-	C–O stretch	
	2921	-	C–H	
	3384	-	O–H	
*Cichorium intybus*	3413.05	-	O–H alcoholic group	[40]
	2922.98	-	Aliphatic C–H group	
	1619.08	-	C=C	
	1384.6	-	C–H	
	1114.28	-	C–O–C	
	-	874.47	N–H	
*Cosmos caudatus*	3364.81	-	O–H	[41]
	2925.49	-	C–H	
	1650.59	-	C=O	
	1384.67	-	C-N	
	1067.62	-	O–H secondary alcohols	
*Cosmos Sulphureus*	-	1643.35	––C==C––	[42]
	-	2980.02	C––H	
	-	3421.72	O––H	
	1637.56	-	––C==C––	
	2981.95, 3748.2	-	C––H	
*Cynara scolymus*	-	538	Ag+ to Ag	[45]
*Dahlia pinnata*	1064 and 3265	-	Aromatic compounds	[46]
	2916	-	C-H stretching of aldehydes	
	673 and 1595	-	Halo-alkanes and bending of C-H bonds	
*Echinacea purpurea*	3,203	-	OH stretching	[49]
	2929 and 2829	-	C–H bonds	
*Echinops* sp.	3395	-	OH stretching frequencies	[50]
	1718	-	C=O vibration of ketonic groups	
	2925	-	C−H stretching mode	
	601	-	Ag–O bond	
*Eclipta alba*	3603 and 3471	-	O–H stretch	[51]
	3379 and 3278	-	Primary and secondary amines and amides	
	2931	-	C–H stretch	
	1064	-	C–N stretch represents aliphatic amines	
*Elephantopus scaber*	1611 to 1400	-	Presence of aromatic rings in the leaf extract.	[52]
	1109	-	Presence of OH groups	
*Erigeron bonariensis*	3376	-	-OH groups of phenolic compounds and -NH stretching of the proteins	[53]
	-	3434	Intensity of Ag	
*Helichrysum graveolens*	2927	-	C–O stretching, free	[60]
	1608	-	C=O stretching	
	1035	-	C–N stretching	
	1417	-	O–H bend	
	-	820	C–O stretching	
	-	606	C–X stretching vibration	
	2358	-	C–H asymmetric stretching	
*Oedera genistifolia*	1117	-	Plant extract	[67]
	1118	-	NP synthesized	
*Spilanthes calva*	3919.31	-	O-H-stretch	[80]
	3435	-	O-H-stretch	
	1412.79	-	C-F stretch	
	1257.83	-	C-F stretch	
*Tagetes erecta*	3401	-	O–H group	[83]
	2940	-	Aromatic compounds	
	1673	-	–C=C– bond	
	1104	-	C–N bond	
*Taraxacum officinale*	3360 to 3400	-	-NH2 in primary aromatic amines and -OH groups	[85]
	2300 to 2990	-	C-H	
	1421	-	C=C	
	1610	-	C=O	
	1063	-	C-OH	
*Tithonia diversifolia*	3398	-	O–H stretching vibrations of polyols	[86]
	1641	-	Stretching vibration of (NH) C O group	
	-	672	N–H	
*Tragopogon Collinus*	3385	-	OH	[88]
	2921	-	NH	
	-	1640	C–O in amide I	
	-	1413	NH2 group in amide II	
*Vernonia cinerea*	1633	-	Amide I, C=O groups	[91]
	3431	-	O–H stretching	
	1515 and 1540	-	–C=C (aromatic ring)	
	1380	-	O–H in-plane bend of phenol	
*Wedelia chinensis*	1022	-	C–O	[92]
	1326	-	C-O-C stretching	
	1696	-	C=O	
Gold NPs				
*Arctium lappa*	3307	-	-OH stretching and the aliphatic methylene group -C-H stretching	[94]
	2151	-	Alkynes group	
	1634	-	Carboxyl stretching	
	-	415, 406, 394 and 383	Metal biomolecules found in the extract	
*Erigeron annuus*	3100, 2850, 2620, 1300, 1100, and 620	-	Extract	[100]
	2900	-	C-H stretching vibration in methylene group	
	1405	-	Hydrocarbons of methylene group	
*Rhanterium epapposum*	1622 to 1630	-	C=O stretching of carbonyl groups	[72]
	-	925 to 553	Stretching of haloalkanes	
*Stevia rebaudiana*	1078	-	Nitrogen–carbon C-N bond stretching of aliphatic amine groups	[104]
	240 and 1634	-	Amides III and II bands of proteins	
	-	1629	Amide I	
Copper NPs				
*Ageratum houstonianum*	3264.96	-	O–H stretch	[159]
	2916.19	-	N+–H stretch	
	2359.9	-	C–H stretching	
	-	1074.64	O-C stretching	
	**-**	667.81	Aromatic H bending	
	**-**	597.86		
*Blumea balsamifera*	3378	-	OH bond of phenolic compound such as flavonoids, tannins, and glycoside	[108]
	1100 and 1700		C-O and C=O	
		610	Cu NPs	
*Eclipta prostrata*	3333	-	Hydroxy group	[109]
	2917	-	Methylene C-H asym./sym. stretch	
	1615	-	Aromatic ring stretch	
	**-**	1610	NH C=O to metals CuNPs	
*Pluchea sericea*	3341	-	O-H stretching	[110]
	2935	-	C-H and N-H bonds	
	1623–1410	-	C=N stretching vibrations	
	1046	-	C=O	
	**-**	622	Cu NPs	
Titanium oxide NPs				
*Ageratina altissima*	3287	**-**	Alcohol, phenols with O-H stretches	[137]
	2922	**-**	Ammonium ions with N-H stretching	
	1645	**-**	Acyclic compound with C-C stretching	
	1537	**-**	Aliphatic of the nitro compound with stretching of N-O	
	1238	**-**	C-O stretching	
	1150	**-**	Alcohol compound with C-O stretching	
*Echinacea purpurea*	1024	**-**	C-O stretching alcohols	[138]
	1385	**-**	C-H rock alkenes	
	1590	**-**	C=C characteristic of saturated hydrocarbons	
	3320	**-**	O-H	
*Sonchus asper*	3937	**-**	OH stretching vibrations	[139]
	3190	**-**	N-H stretching	
	2851	**-**	Symmetric CH2 stretching bands	
	2600	**-**	H bonded NH vibrations	
	**-**	1000 and 500	Ti-O-Ti linkage in TiO_2_	
Copper oxide NPs				
*Eupatorium odoratum*	3976	**-**	Adsorbed water molecules	[136]
	3406	**-**	-OH stretching vibrations of phenolic group	
	1520	**-**	C‚ C stretch in aromatic rings	
	1420	**-**	O-H bend of polyphenol	
	**-**	1121	Cu-OH vibrations	
	**-**	815 and 613	-CH bending vibrations	
	-	653 and 610	Cu-O signals	

Note: NPs: nanoparticles;—: not applicable.

## Data Availability

The data presented in this study are available on request from the corresponding authors.
